# Coriolus Versicolor Downregulates TLR4/NF-κB Signaling Cascade in Dinitrobenzenesulfonic Acid-Treated Mice: A Possible Mechanism for the Anti-Colitis Effect

**DOI:** 10.3390/antiox11020406

**Published:** 2022-02-17

**Authors:** Daniela Impellizzeri, Roberta Fusco, Tiziana Genovese, Marika Cordaro, Ramona D’Amico, Angela Trovato Salinaro, Maria Laura Ontario, Sergio Modafferi, Salvatore Cuzzocrea, Rosanna Di Paola, Vittorio Calabrese, Rosalba Siracusa

**Affiliations:** 1 Department of Chemical, Biological, Pharmaceutical and Environmental Sciences, University of Messina, 98166 Messina, Italy; dimpellizzeri@unime.it (D.I.); tiziana.genovese@unime.it (T.G.); rdamico@unime.it (R.D.); rsiracusa@unime.it (R.S.); 2 Department of Clinical and Experimental Medicine, University of Messina, 98125 Messina, Italy; rfusco@unime.it; 3 Department of Biomedical, Dental and Morphological and Functional Imaging, University of Messina, 98125 Messina, Italy; marika.cordaro@unime.it; 4 Department of Biomedical and Biotechnological Sciences, University of Catania, 95125 Catania, Italy; trovato@unict.it (A.T.S.); marialaura.ontario@ontariosrl.it (M.L.O.); sergio.modafferi@studium.unict.it (S.M.); calabres@unict.it (V.C.); 5 Department of Pharmacological and Physiological Science, Saint Louis University School of Medicine, Saint Louis, MO 63104, USA; 6 Department of Veterinary Science, University of Messina, 98168 Messina, Italy

**Keywords:** inflammatory bowel diseases, inflammation, oxidative stress, natural compounds, TLR4

## Abstract

Inflammatory bowel diseases (IBDs) are disorders characterized by chronic inflammation of the intestinal tract. The focus of the present study was to examine the effect of the fungus *Coriolus versicolor* (*CV*), underlining its correlation with Toll-like receptors 4 (TLR4) and nuclear factor erythroid 2-related factor 2 (Nrf2); we aim to evaluate its anti-inflammatory and antioxidant effect in mice exposed to experimental colitis. The model was induced in mice by colon instillation of dinitrobenzenesulfonic acid (DNBS), *CV* was administered orally (200 mg per kg) daily for 4 days. On day 4, the animals were killed, and the tissues collected for histological, biochemical, and molecular analyses. Four days after DNBS administration, CC motif chemokine ligand 2 (CCL2), prostaglandin E2 (PGE2), interleukin-1β (IL-1β), and tumor necrosis factor-α (TNF-α) production increased in association with damage to the colon. Neutrophil infiltration, as assessed by myeloperoxidase (MPO) activity, in the mucosa was associated with overexpression of P-selectin and intercellular adhesion molecule 1 (ICAM1). Immunohistochemistry for nitrotyrosine and poly-(ADP-Ribose)-polymerase (PARP) showed evident stain in the inflamed colon. Treatment with *CV* significantly reduced the appearance of colon changes and weight loss. These effects were associated with a remarkable ability of *CV* to reduce the expression of TLR4 and modulate the pathway of nuclear factor kappa-light-chain-enhancer of activated B cells (NF-kB). This improved the colon architecture, reduced MPO activity, the release of proinflammatory cytokines, the presence of nitrotyrosine, and the hyperactivation of PARP, as well as the up-regulation of P-selectin and ICAM1. Furthermore, we studied the action of *CV* on the Nrf2/HO-1 pathway, which is important for maintaining redox balance, demonstrating that *CV* by significantly increasing both enzymes is able to counteract the oxidative stress induced by DNBS. Taken together, our results clearly show that this natural compound can be considered as a possible dietary supplement against colitis.

## 1. Introduction

Inflammatory bowel disease (IBD) is a term used to call ailments involving chronic inflammation of the digestive tract. There are mainly two types of IBD: ulcerative colitis and Crohn’s disease. Ulcerative colitis is characterized by inflammation and ulcer formation along the superficial lining of the colon and rectum. Crohn’s disease, on the other hand, is characterized by inflammation of the lining of the digestive tract that can often comprise the deeper layers of this tract [1].

People of different ages can get IBD, but it is usually diagnosed between the ages of 15 and 40 [2].

Both types of IBD are generally characterized by diarrhea, rectal bleeding, abdominal pain, fatigue, and weight loss. IBD can therefore be debilitating and sometimes lead to life-threatening complications. The causes of IBD are still unclear, but it is thought to be caused by a combination of factors, including: genetics, immune system changes, and lifestyle [3].

What has certainly been observed over the years is that both types of IBD are characterized by an increase in pro-inflammatory cytokines, chemokines, and adhesion molecules that increase the damage to the barrier function and perpetuate the inflammatory process [4,5]. To confirm this there are studies conducted on mucous biopsies of patients with IBD [6].

Furthermore, recently, various studies are focusing on reactive nitrogen and oxygen species (RNS and ROS) as etiological causes for IBD [7]. The intestine represents the main place of origin of pro-oxidant molecules, due to a surplus of food constituents, microbes and interactions between immune cells [7]. Furthermore, a reduction in antioxidant capacity has been highlighted in patients with IBD and in asymptomatic subjects [8]. To counteract the increase in oxidative stress, intestinal cells require enzymatic and non-enzymatic antioxidant systems [9]. Therefore, oxidative stress concomitant with immune activation and inflammation could contribute to the tissue damage and fibrosis that characterize intestinal diseases [10].

There is currently no cure for ulcerative colitis or Crohn’s disease. Most existing treatments aim to relieve symptoms and prevent them from returning and include specific diets, lifestyle changes, medications (sulfasalazine; corticosteroids; immunosuppressive agents; and several biologics, for example anti-TNF-α antibodies) and, in the most serious cases, surgery [11,12,13,14].

Since oxidative stress and inflammation contribute to tissue damage during colitis, the administration of natural compounds with antioxidant and anti-inflammatory activity has recently been proposed as a treatment for IBD [13,15]. Mushrooms have been used in traditional medicine for many years [16]. Several studies have investigated the medicinal action that mushrooms and their extracts possess, including anti-tumor, immunomodulatory, anti-oxidant, anti-inflammatory, antiviral, antibacterial, and hepatoprotective effects [17].

Mushrooms have been shown to stimulate the host’s immune system. This appears to be due to the high content of β-glucans, which activate different types of immune cells and stimulate the cytokine response [17,18].

The polysaccharopeptides produced by *Coriolus versicolor* (*CV*) are widely used to integrate chemotherapy and radiotherapy of cancer and infectious diseases [19]. Over the years, *CV* has been shown to have both anticancer and immunomodulatory properties as well as anti-inflammatory and antioxidant properties. In a mouse model of Alzheimer’s disease, *CV* has been shown to improve spatial memory by activating antioxidants such as superoxide dismutase (SOD) and catalase (CAT) and inhibiting pro-inflammatory cytokines [20]. In our recent study, *CV*’s ability to reduce inflammatory processes induced by traumatic brain injury (TBI) and inducing neurodegeneration has emerged. From the characterization of *CV,* it emerged that the main components of this fungus are hexadecanoic acid, hexadecane, and vanillic acid which are probably responsible for the anti-inflammatory and anti-oxidant action observed in an experimental model of TBI [21].

Based on the data in the literature, in the present study, we used a model of colitis induced by dinitrobenzenesulphonic acid (DNBS) to evaluate the effects of the fungus *CV* on the regulation of inflammatory processes and oxidative stress and in particular the modulation of Toll-like receptors 4 (TLR4) and nuclear factor erythroid 2-related factor 2/heme oxygenase 1 (Nrf2/HO-1) pathway.

## 2. Materials and Methods

### 2.1. Animals

CD1 male mice (25–30 g) were obtained from Envigo (Milan, Italy) and were housed in a sterile environment with water and food suitable for mice. The cages in which the animals were kept were in rooms with a temperature of 22 ± 1 °C and day/night cycles of 12 h each. They were kept in a controlled location and received food and water ad libitum. The University of Messina Review Board for animal care (OPBA approval number 89126.24/2021) approved the study. All in vivo experiments followed the new directives of the USA, Europe, Italy, and the ARRIVE guidelines.

### 2.2. Colitis Induction and Treatment

The intra-rectal injection of DNBS (4 mg in 100 μL of 50% ethanol per mouse) was executed on day 0 for induction of colitis as previously reported [22].

*CV* biomass including mycelium and primordia of the mushroom, offered by Mycology Research Laboratories Ltd. (MRL, Luton, UK), as the product commercially existing, was used for investigation. Ideal quantity (200 mg/kg) was chosen according to the dose used in clinical trials with cancer or human papilloma virus (HPV) patients (3 g/day) [23], a regimen also confirmed by studies in rat and mice [21,24,25]. After 4 days to colitis induction, mice were sacrificed and the colon was harvested, opened, rinsed, and processed for histology and biochemical analyses.

### 2.3. Experimental Design

All animals were randomized in the indicated groups (10 mice for each group):

1. Sham + vehicle: vehicle solution (saline) was administered daily orally and animals were sacrificed on day 4 after intrarectal administration of vehicle (100 μL of 50% ethanol);

2. Sham + *CV*: mice were subjected to the procedures as above and animals were treated orally with *CV* (data are not shown in all results as no significant differences were observed with the Sham + vehicle);

3. DNBS: vehicle was administered daily orally, and animals were sacrificed on day 4 after administration of DNBS.

4. DNBS + *CV*: mice were administered intrarectal DNBS as described above and *CV* (200 mg/kg dissolved in saline, orally) was administered every 24 h, starting 1 h after administration of DNBS and animals were sacrificed on day 4 after colitis induction.

### 2.4. Body Weight and Macroscopic Analysis of Colon Damage

Mice were weighed all day, from day 0 until the day of sacrifice. The colon tissue was collected and untied by a longitudinal incision. Two impartial observers reported the severity of colon damage following standardized criteria as described above [26]. Briefly, macroscopic colon damage was evaluated and scored according to the following criteria: 0 = no damage; 1 = localized hyperemia without ulcers; 2 = linear ulcers with no important inflammation; 3 = linear ulcers with inflammation at one site; 4 = two or more major sites of inflammation and ulceration covering more than 1 cm along the length of the colon; and 5–8 = one point is added for each cm of ulceration beyond an initial 2 cm.

### 2.5. Histological Evaluation

Colon were treated with hematoxylin and eosin (H&E) staining for histological valuation; the semi-quantitative assessment of the damage was evaluated according to a score ranging from 0 to 4 as described in literature [27], in a blinded mode by two qualified pathologists using a Leica DM6 microscope (Leica Microsystems SpA, Milan, Italy) associated with Leica LAS X Navigator software (Leica Microsystems SpA, Milan, Italy). Briefly the assigned score corresponded to the following characteristics: 0 = no damage; 1 = mild damage with focal epithelial edema and necrosis; 2 = moderate damage with widespread swelling and necrosis of the villi; 3 = severe damage with necrosis and presence of neutrophil infiltrate in the submucosa; 4 = very severe damage with diffuse necrosis and massive infiltrate of neutrophils and hemorrhage.

### 2.6. Malondialdehyde (MDA) Assay

As previously reported, MDA levels, a marker of lipid peroxidation, were determined in colon tissue 4 days after DNBS administration [28,29]. Briefly, the colon was homogenized in a 1.15% KCl solution. About 100 μL of homogenate was added to a reaction mixture containing 8.1% sodium dodecyl sulfate, 20% acetic acid (pH 3.5), 0.8% thiobarbituric acid, and distilled water. The samples were boiled for 1 h at 95 °C and centrifuged at 3000× *g* for 10 min. The absorbance was measured by spectrophotometer at 532 nm.

### 2.7. Myeloperoxidase (MPO) Activity

MPO activity, an indicator of neutrophil infiltration, was determined by spectrophotometric assay with tetramethylbenzidine as the substrate. The collected colons were homogenized in a solution containing 0.5% hexadecyl-trimethyl-ammonium bromide dissolved in potassium phosphate buffer (10 mM pH 7) and subsequently centrifuged for 30 min at 20,000× *g* at 4 °C. Part of the supernatant was then allowed to react with a solution of tetramethylbenzidine (1.6 mM) and H_2_O_2_ (0.1 mM). A reading at 650 nm was performed using a spectrophotometer. MPO activity was expressed in unit of MPO/g of wet tissue and was measured as the quantity of enzyme degrading 1 µM of peroxide per min at 37 °C [29,30].

### 2.8. Immunohistochemical Evaluation of CD4^+^, CD8^+^, Cell Adhesion Molecules (ICAM-1, P-Selectin), Poly-(ADP-ribose polymerase) (PARP), and Nitrotyrosine

Immunohistochemical examination was performed as previously described [31], 4 days after DNBS administration. The sections were incubated overnight with the following primary antibodies: anti-CD4^+^ (Santa Cruz Biotechnology (SCB), 1:100 in PBS, *v/v*, sc-19641, D.B.A, Milan, Italy); anti-CD8^+^ (SCB, 1:100 in PBS, *v/v*, sc-1177, D.B.A, Milan, Italy); anti-ICAM-1 (SCB, 1:100 in PBS, *v/v*, sc-107, D.B.A, Milan, Italy), anti-P-selectin (1:100 in PBS, *v/v*, sc-8419 SCB, D.B.A, Milan, Italy), anti-PARP (1:100 in PBS, *v/v*, sc-8007 SCB, D.B.A, Milan, Italy), and anti-nitrotyrosine (1:200 in PBS, *v/v*, Millipore, D.B.A, Milan, Italy). All slices were washed with PBS and then treated as previously reported [32]. Briefly, slices were incubated with universal secondary antibody. Specific labeling was detected with a biotin- conjugated goat anti-rabbit IgG and avidin-biotin peroxidase complex (Vector).

Five stained sections from each mouse were scored in a blinded fashion and observed using a Leica DM6 microscope (Leica Microsystems SpA, Milan, Italy) following a typical procedure [33]. The histogram profile is related to the positive pixel intensity value obtained.

### 2.9. Western Blots Analyses

Cytosolic and nuclear proteins were extracted from colon tissues as previously described [28]. The behind primary antibodies were used: anti-TLR4 (SCB, 1:500, #sc293072, D.B.A, Milan, Italy); anti-Myd88 (SCB; 1:500, #sc-74532, D.B.A, Milan, Italy); anti-IKB-α (SCB, 1:500, #sc-4094, D.B.A, Milan, Italy), anti-NF-kB (SCB; 1:500 #sc8008, D.B.A, Milan, Italy), anti-Nrf2 (SCB, 1:500; #A-10:sc-365949; D.B.A, Milan, Italy); anti-HO-1 (SCB, 1:500; #sc-136960; D.B.A, Milan, Italy), in PBS with 5% *w/v* non-fat dried milk and 0.1% Tween-20 at 4 °C O/N. Membranes were incubated with peroxidase-conjugated bovine anti-mouse IgG secondary antibody or peroxidase-conjugated goat anti-rabbit IgG (Jackson ImmunoResearch, West Grove, PA, USA; 1:2000) for 1 h at room temperature. Anti-β-actin or anti-lamin A/C (D.B.A, Milan, Italy) antibodies were used as controls. Protein expression was analyzed as previously reported [34].

### 2.10. Cytokine Measurements

In the colon tissues obtained after sacrifice, TNF-α (Ray Bio ELISA Kit Mouse TNF-alpha, Norcross, GA, USA), IL-1β, CCL2, and PGE2 (R&D Systems, Milan, Italy) levels were evaluated using a colorimetric commercial kit [22].

### 2.11. Statistical Analysis

All values in the figures and text are expressed as mean ± standard deviation (SD) of N observations. For the in vivo studies, N represents the number of animals studied. In experiments involving histology, the figures shown are representative of at least three experiments performed on different days on tissue sections collected from all animals in each group. The results were analyzed by one-way ANOVA followed by a Bonferroni post hoc test for multiple comparisons. A *p*-value of less than 0.05 was considered significant.

## 3. Results

### 3.1. Effects of CV on Macroscopic Changes and Body Weight

**Figure 1:**
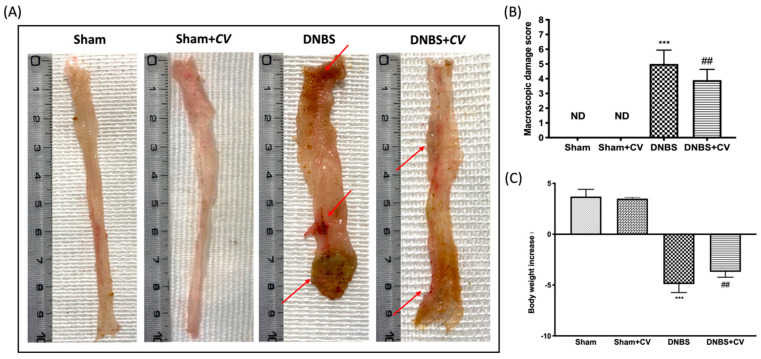
The effects of *CV* on macroscopic damage and body weight after DNBS-injection. Macroscopic changes in Sham, Sham+*CV*, DNBS, DNBS+*CV* groups (**A**). Macroscopic damage score was performed by two independent observers (**B**). Body weight increase (**C**). Values = means ± SD of 10 animals in each group; *** *p* < 0.001 vs. Sham; ## *p* < 0.01 vs. DNBS.

Macroscopic inspection of the colon showed no changes in the Sham and Sham+*CV* groups mice. Animals treated with DNBS, on the other hand, showed a flaccid colon with liquid stools and in some cases, ulcerations with mucosal congestion. Oral treatment with *CV* significantly reduced tissue inflammation and macroscopic signs (Figure 1A,B). Diarrhea observed mainly in mice of the DNBS group also led to a significant reduction in the body weight of these animals compared to the Sham group, while treatment with *CV* was able to restore the weight loss induced by DNBS (Figure 1C).

### 3.2. Effects of CV on Histological Colon Injury

**Figure 2:**
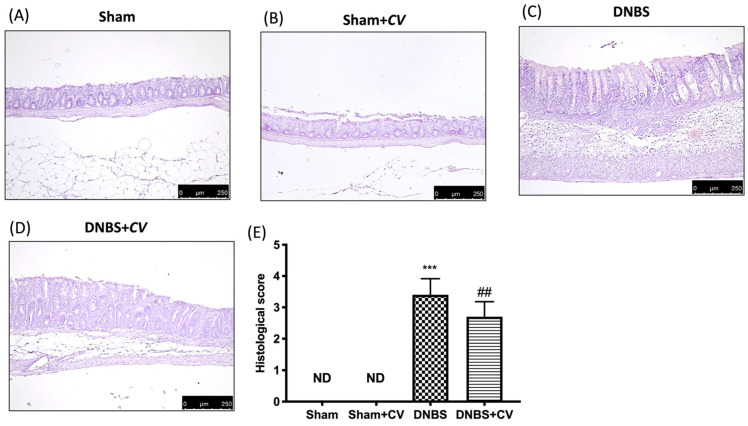
The effects of *CV* on microscopic damage after DNBS-injection. Histological analysis was evaluated in Sham (**A**), Sham+*CV* (**B**), DNBS (**C**), DNBS+*CV* (**D**). The histological score was measured (**E**). Images are figurative of at least three independent experiments. Values = means ± SD of 5 animals in each group; *** *p* < 0.001 vs. Sham; ## *p* < 0.01 vs. DNBS.

Microscopic analysis carried out on sections of colon tissue collected from mice treated with DNBS and stained with H&E showed infiltration of inflammatory cells (mostly neutrophils), necrosis and edema (Figure 2C,E), compared to the tissues of the Sham and Sham+*CV* groups in which no ulcer formation was observed (Figure 2A,B,D). Oral treatment with *CV* reduced histological changes by reducing inflammation and the severity of colitis (Figure 2D,E).

### 3.3. Effects of CV on Inflammatory Pathway

**Figure 3:**
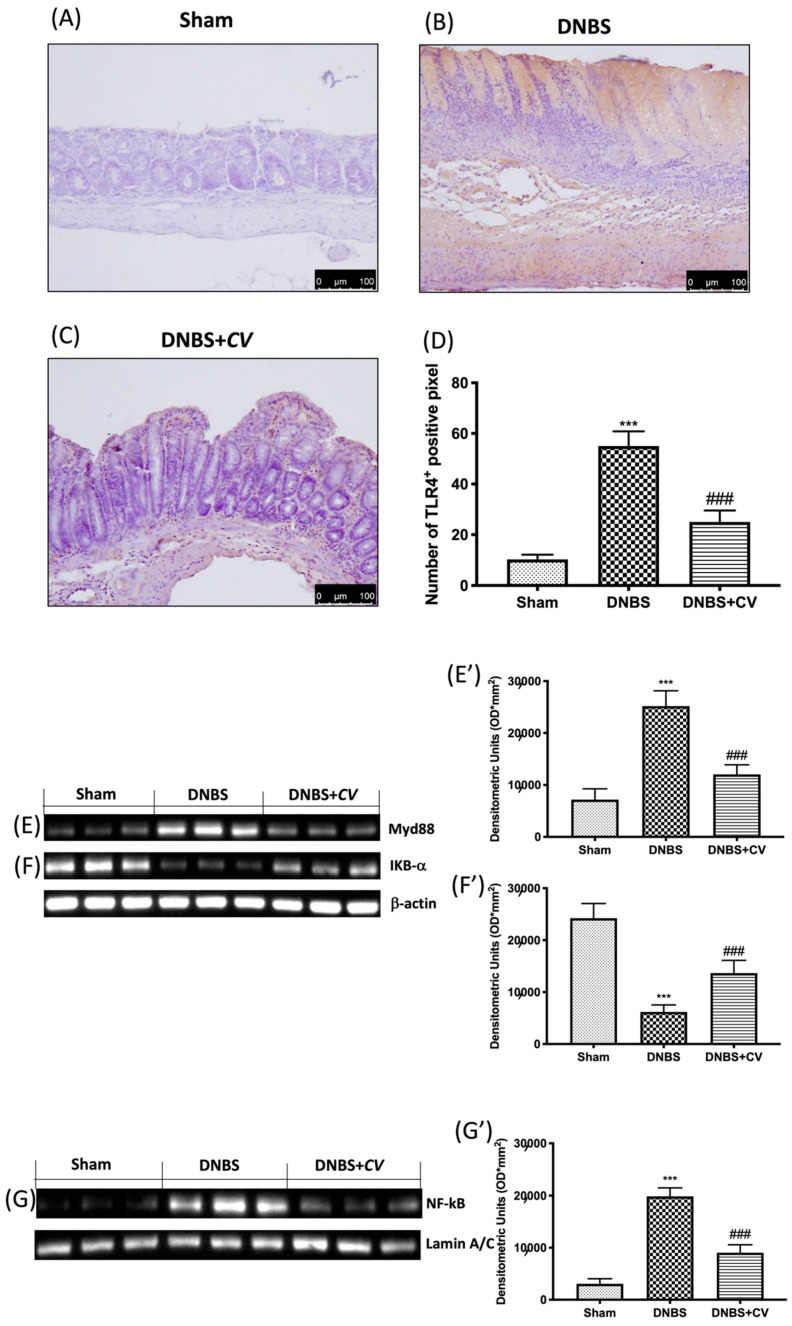
The effects of *CV* on TLR4, Myd88 and NF-kB pathway expression after DNBS-injection. Immunohistochemistry for TLR4 was evaluated in Sham (**A**), DNBS (**B**) and DNBS+*CV* (**C**). The results are expressed as number of TLR4^+^ positive pixel (**D**). Images are figurative of at least three independent experiments. Western blots for Myd88, IKB-α, and NF-kB. Representative Western blots for cytoplasmic Myd88 (**E**,**E’**), IKB-α degradation (**F**,**F’**), nuclear NF-kB translocation (**G**,**G’**) expression were performed. A demonstrative blot of lysates (5 animals/group), with a densitometric analysis for all animals, is shown (**E**’–**G’**). Values = means ± SD of 5 animals in each group. *** *p* < 0.001 vs. Sham; ### *p* < 0.001 vs. DNBS.

In order to study how *CV* acted on inflammatory processes we evaluated the variations of TLR4 and consequently the expression of Myd88 and NF-kB pathway. By the immunohistochemical analysis we observed an increase in the expression of TLR4 in the colon sections taken from the mice treated with DNBS (Figure 3B,D), compared to the control group (Figure 3A,D). On the contrary, treatment with *CV* significantly limited the expression of this receptor (Figure 3C,D). Subsequently, owing to Western blot analyses, we observed how the activation of TLR4 also led to changes in the Myd88 (Figure 3E,E’), IKB-α (Figure 3F,F’), and NF-kB (Figure 3G,G’) proteins. In particular, increase in the expression of Myd88, the degradation of IKB-α, and the consequent nuclear translocation of NF-kB were observed in the DNBS group, while the treatment with *CV* was able to prevent this inflammatory phenomena.

### 3.4. Effects of CV on MPO, MDA, and Pro-Inflammatory Mediators

**Figure 4:**
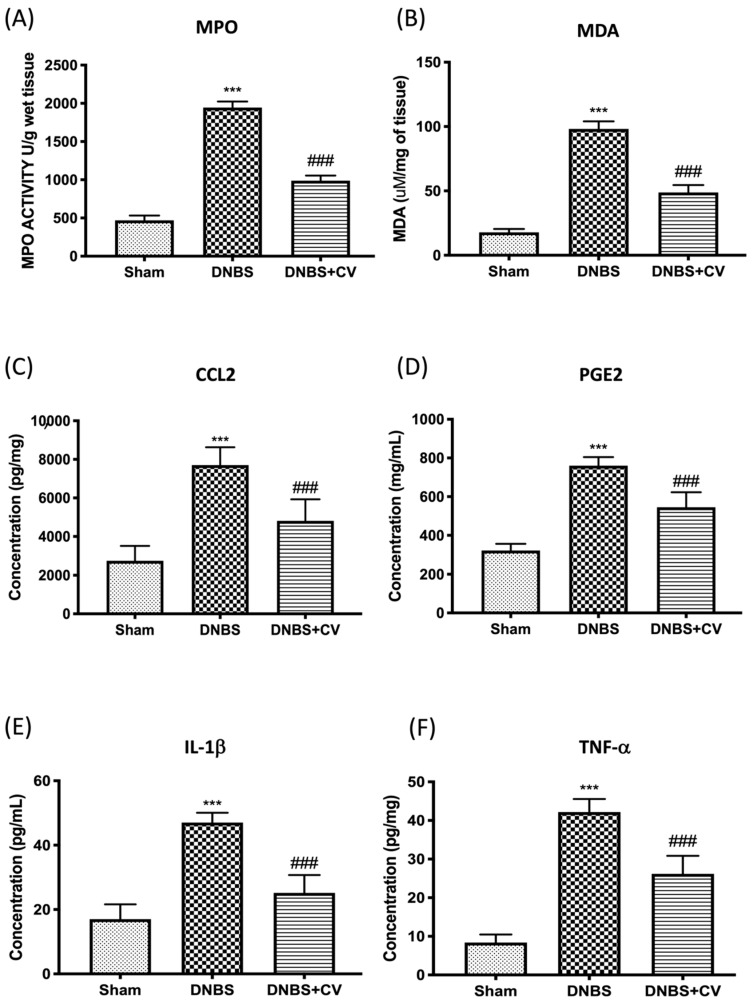
The effects of *CV* on MPO, MDA, CCL2, PGE2, IL-1β, and TNF-α after DNBS-injection. MPO (**A**) and MDA (**B**) assays were performed for neutrophil infiltration and lipid peroxidation. MPO (**A**) and MDA (**B**) assays were performed for neutrophil infiltration and lipid peroxidation. The concentrations of CCL2 (**C**), PGE2 (**D**), IL-1β (**E**), and TNF-α (**F**) in the colon collected after 4 days from the induction of colitis were evaluated using ELISA kits. Values = means ± SD of 5 animals in each group; *** *p* < 0.001 vs. Sham; ### *p* < 0.001 vs. DNBS.

Colon damage induced by DNBS was also illustrated by the increase in neutrophilic infiltration in the colon measured by the activity of MPO (Figure 4A) and by the increase in MDA levels (Figure 4B), compared to the control group. Furthermore, by ELISA tests we measured the levels of other pro-inflammatory mediators involved in inflammation of the colon such as CCL2 (Figure 4C), PGE2 (Figure 4D), IL-1β (Figure 4E), and TNF-α (Figure 4F). The results showed a significant increase of these mediators in the tissues of the group DNBS, compared to the Sham group. Oral treatment with *CV* significantly reduced the accumulation of neutrophils in the colon, lipid peroxidation, the levels of the pro-inflammatory mediators CCL2, PGE2, and of the cytokines IL-1β and TNF-α.

### 3.5. Effects of CV on CD4^+^, CD8^+^, and Adhesion Molecules

CD4^+^ and CD8^+^ T cells are also important mediators of inflammation in the colon, which together with cytokines lead to an increase in adhesion molecules such as P-selectin and ICAM1.

**Figure 5:**
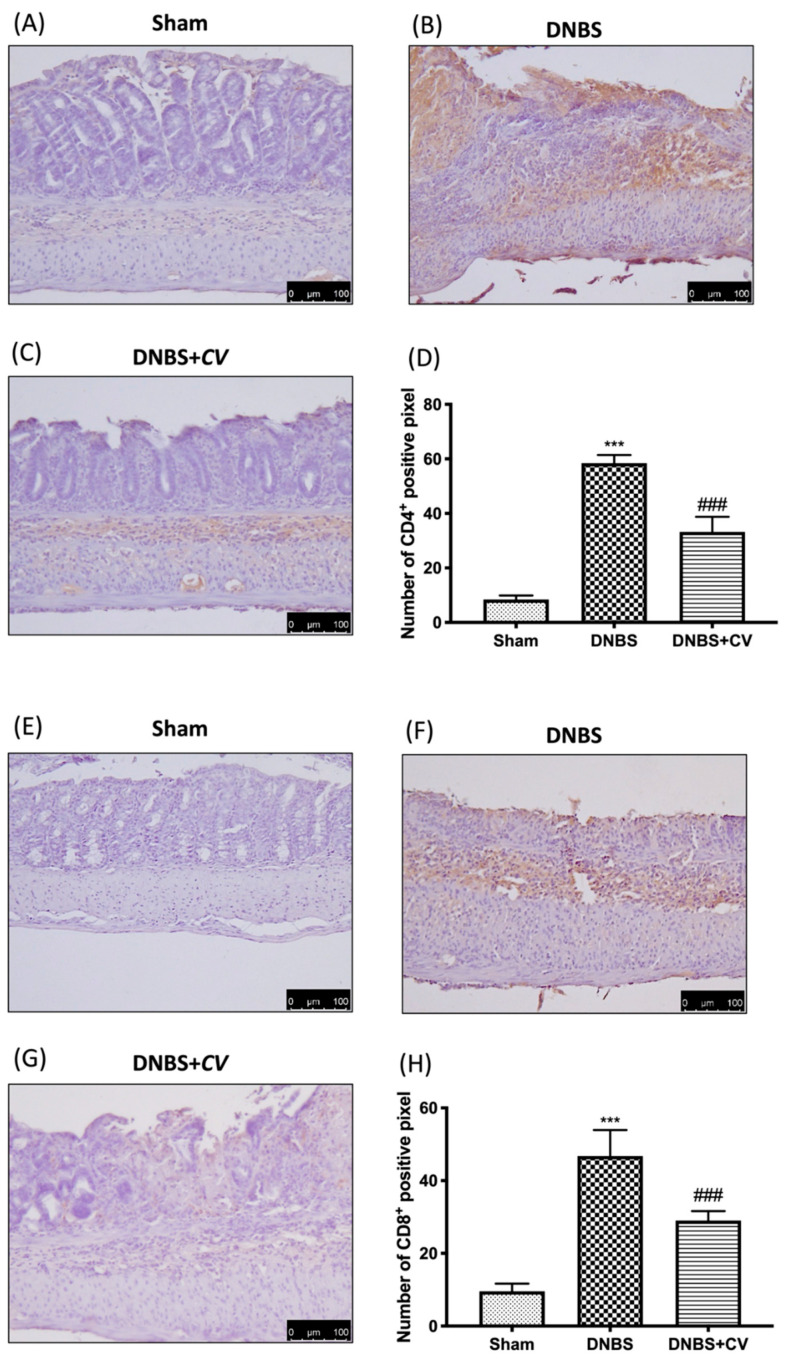
The effects of *CV* on CD4^+^ and CD8^+^ expression after DNBS-injection. Immunohistochemistry for CD4+ was evaluated in Sham (**A**), DNBS (**B**) and DNBS+*CV* (**C**). The results are expressed as number of CD4^+^ positive pixel (**D**). The same analysis was performed for CD8^+^ on the colon sections of the Sham (**E**), DNBS (**F**), DNBS+*CV* (**G**) groups. The results are expressed as number of CD8^+^ positive pixel (**H**). The Images are figurative of at least three independent experiments. Values = means ± SD of 5 animals in each group. *** *p* < 0.001 vs. Sham; ### *p* < 0.001 vs. DNBS.

**Figure 6:**
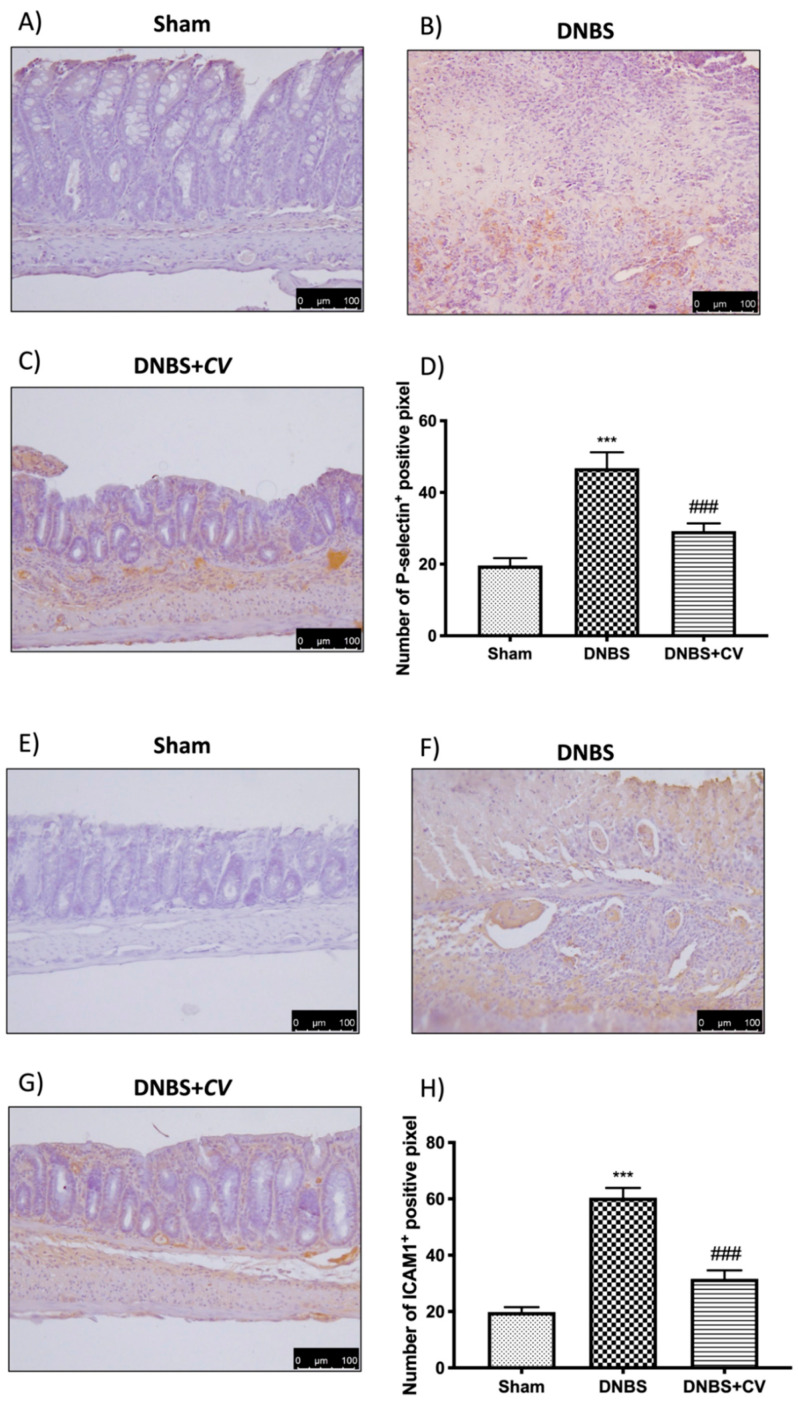
The effects of *CV* on P-selectin and ICAM1 expression after DNBS-injection. Immunohistochemistry for P-selectin was evaluated in Sham (**A**), DNBS (**B**) and DNBS + *CV* (**C**). The results are expressed as number of P-selectin^+^ positive pixel (**D**). The same analysis was performed for ICAM1 on the colon sections of the Sham (**E**), DNBS (**F**), DNBS+*CV* (**G**) groups. The results are expressed as number of ICAM1^+^ positive pixel (**H**). The Images are figurative of at least three independent experiments. Values = means ± SD of 5 animals in each group. *** *p* < 0.001 vs. Sham; ### *p* < 0.001 vs. DNBS.

Significant positive staining was observed for CD4^+^ and CD8^+^ in colon tissues of animals treated with DNBS, compared to the Sham group where no positive staining was evident. *CV* treatment reduced positive staining for both CD4^+^ and CD8^+^ (Figure 5A–D for CD4^+^; Figure 5E–H for CD8^+^). The increase in these cells led to an increase in the expression of P-selectin and ICAM1 in the colon of mice of the DNBS group, compared to the control group. Moreover, in this case the administration of *CV* led to a reduction of the positive staining for both adhesion molecules (Figure 6A–D for P-selectin; Figure 6E–H for ICAM1).

### 3.6. Effects of CV on Nitrotyrosine and PARP Expression

**Figure 7:**
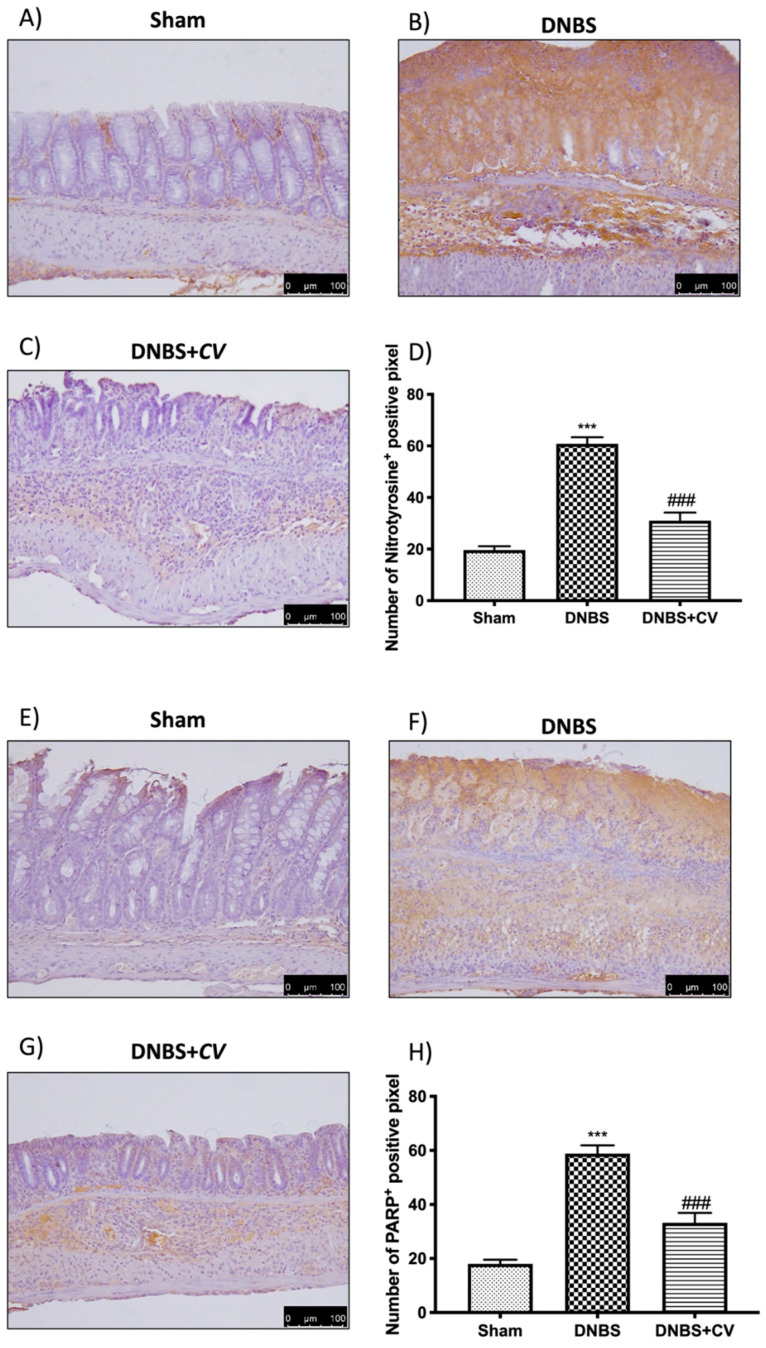
The effects of *CV* on nitrotyrosine and PARP expression after DNBS-injection. Immunohistochemistry for nitrotyrosine was evaluated in Sham (**A**), DNBS (**B**) and DNBS + *CV* (**C**). The results are expressed as number of nitrotyrosine^+^ positive pixel (**D**). The same analysis was performed for PARP on the colon sections of the Sham (**E**), DNBS (**F**), DNBS + *CV* (**G**) groups. The results are expressed as number of PARP^+^ positive pixel (**H**). The images are figurative of at least three independent experiments. Values = means ± SD of 5 animals in each group. *** *p* < 0.001 vs. Sham; ### *p* < 0.001 vs. DNBS.

To study the effect of *CV* on nitrosative stress and on PARP hyperactivation we performed an immunohistochemical analysis on the colon sections. This analysis showed a significant positive staining in the colon of the mice of the DNBS group for both nitrotyrosine and PARP, unlike the Sham group where no staining was highlighted. Treatment with *CV* was able to reduce nitrosative stress and PARP hyperactivation in the colon showing less positive staining than the vehicle group (Figure 7A–D for Nitrotyrosine; Figure 7E–H for PARP).

### 3.7. Effects of CV on Nrf2/HO-1 Pathway

**Figure 8:**
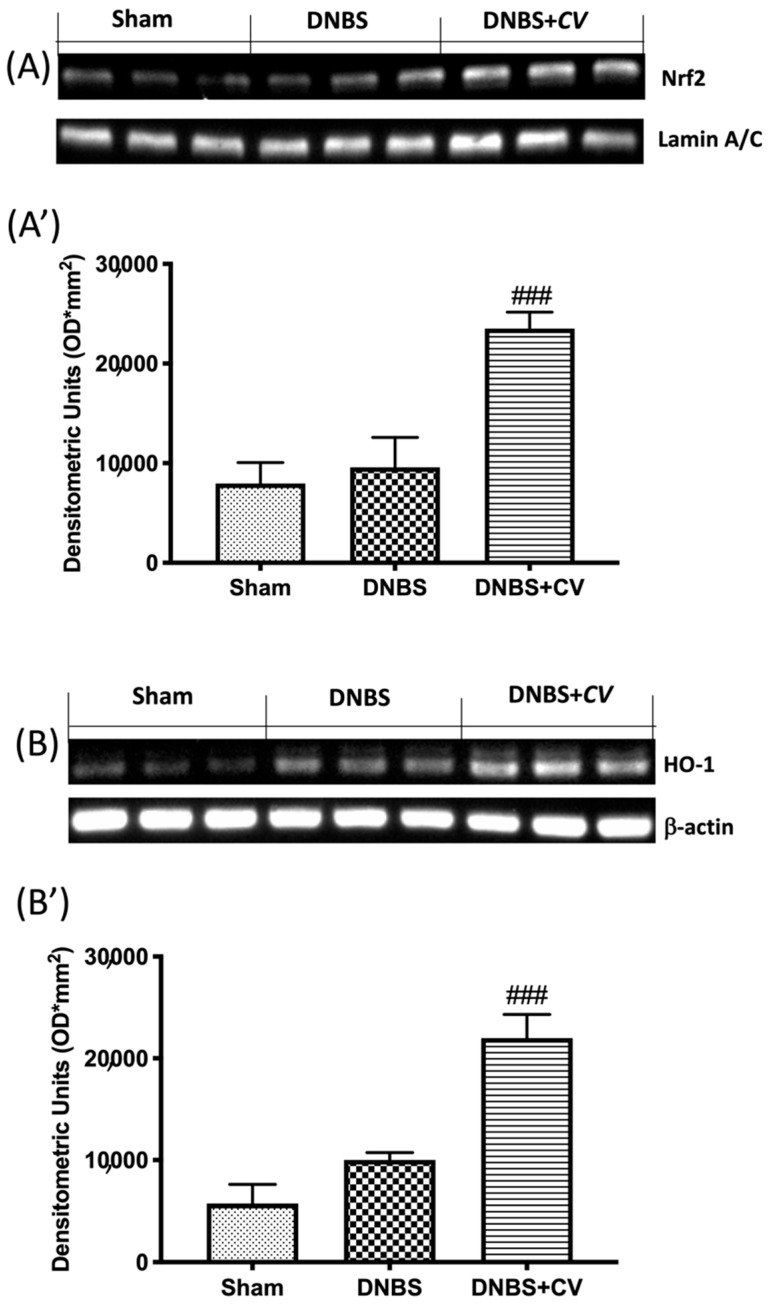
The effects of *CV* on Nrf2/HO-1 pathway expression after DNBS-injection. Representative Western blots for nuclear Nrf2 (**A**,**A’**) and cytoplasmic HO-1 (**B**,**B’**) expression were performed. A demonstrative blot of lysates (5 animals/group), with a densitometric analysis for all animals, is shown (**A’**,**B’**). Values = means ± SD of 5 animals in each group. ### *p* < 0.001 vs. DNBS.

As ROS are known to play an important role in potentiating inflammation, we assessed by Western blot analysis whether *CV* treatment can modulate oxidative processes through the Nrf2/HO-1 pathway. On day 4, *CV* administration significantly increased Nrf2 expression (Figure 8A,A’). Furthermore, based on the observed increase in Nrf2 expression, we also evaluated the expression of HO-1, another important enzyme involved in the oxidative stress pathway. The results obtained from our experiments also showed a significant increase in HO-1 expression after treatment with *CV* (Figure 8B,B’).

## 4. Discussions

IBD are inflammatory bowel diseases characterized by dysfunction of the immune response of the mucosa, abnormal production of pro-inflammatory cytokines such as TNF-α and IL-1, increased expression of adhesion molecules and infiltrated cell, imbalance of the redox balance that eventually led to epithelial cell apoptosis and mucosal damage [35,36,37]. A mechanism responsible for the development of the inflammatory reaction in colitis is the activation of TLR4. The increase of the expression of TLR4 and the increase of the production of CCL2, COX-2, PGE2, and TNF-α compromise the regeneration of the mucosa, with consequent tissue damage that over time can lead to the development of cancer of the colon [38,39]. Recent studies have highlighted the ability of extracts from *CV* to act as anti-inflammatory agents with cytotoxic properties against endothelial cells and breast cancer cells. This protective action has been linked to the ability of these extracts to reduce the activation of TLR4 and consequently limit the inflammatory response [40,41,42]. In addition, dietary interventions with *CV* have been shown to modulate mitochondrial production of ROS and oxidative damage repair in numerous chronic diseases [25,43].

Therefore, our hypothesis was that *CV* could act by modulating TLR4 on inflammatory processes and oxidative stress characteristic of IBD in an animal model of DNBS-induced colitis. To understand the molecular mechanisms involved in the effects of *CV* on inflammation of the DNBS colon, we studied the expression of TLR4 by immunohistochemical analysis. A significant increase in TLR4 expression was observed in mice treated with DNBS compared to control mice, while oral administration of *CV* was able to significantly reduce the expression of this receptor. To confirm the involvement of TLR4 and the ability of *CV* to interfere with the cascade of the TLR4 signal, we evaluated the expression of Myd88 and the NF-kB pathway by Western blot analysis. The results obtained showed a significant increase of Myd88 in mice in the vehicle group which led to IKB-α degradation and nuclear translocation to NF-kB. Treatment with *CV* has significantly limited these effects.

In addition, during inflammation of the colon several pro-inflammatory mediators are released following transcriptional induction of the NF-kB pathway; therefore, we checked whether treatment with *CV* modulated the secretion of pro-inflammatory agents such as TNF-α, IL-1β, CCL2, and PGE2 by measuring their levels using an ELISA kit. The results we obtained showed that the activation of the NF-kB pathway led to the significant increase of these pro-inflammatory mediators, while the treatment with *CV* was able to reduce the levels of TNF-α, IL-1β, CCL2, and PGE2 significantly. Other known mediators of inflammation in IBD are CD4^+^ and CD8^+^ T cells. These active cells were found in the peripheral blood and intestinal mucosa of subjects with IBD [44,45,46]. An abnormal immune response with excessive activation of CD4^+^ and CD8^+^ T cells was also found in our study in mice treated with DNBS. Treatment with *CV* was able to limit the activation of these cells, contributing together to the reduction of the inflammatory process. The increase in pro-inflammatory mediators at the intestinal level in turn causes an increase in the expression of adhesion molecules such as selectins and addressins that stimulate the infiltration of leukocytes into the intestinal mucosa [47]. In accordance with the data in the literature, we observed significantly increased levels of P-Selectin and ICAM1 in the group of animals treated with DNBS, probably due to the increase in intestinal lesion that was highlighted by our macroscopic and microscopic analyses. Treatment with *CV* significantly abolished the expression of both proteins. In addition, the lower expression of adhesion molecules in the colon tissue of mice treated with *CV* is, at least in part, related to the reduction of polymorphonuclear number (PMN), as shown by the activity of MPO, and the reduction of tissue damage and ulcer formation. As noted in several studies, tissue damage to the colon is not only due to an abnormal response of the immune system and therefore to the activation of inflammatory processes, but also to an increase in the release of ROS and redox imbalance [48]. In particular, the increase in ROS levels can negatively affect proteins, nucleic acids, and lipids through the instigation of fragmentation products that lead to enzymatic alterations, release of lipid peroxides, and formation of DNA strand break products [49]. Being able to act on the levels of ROS not only means restoring the redox balance between antioxidant enzymes and not, but also decreasing nitrosative stress and hyperactivation of Poly ADP-ribose (PAR) [50]. Here, we have clearly shown that *CV* inhibits the appearance of nitrotyrosine and PARP staining in the inflamed colon, suggesting that *CV* can act through several pathways in addition to the inflammatory one. In particular, we studied an important antioxidant pathway, Nrf2/HO-1, which seems to be related to NF-kB signaling [51]. Nrf2 is the main transcriptional regulator of antioxidant proteins that translates into the nucleus and promotes the expression of HO-1 after cell damage [52,53]. Our data have highlighted the ability of *CV* to activate Nrf2 and consequently also HO-1 and therefore act on antioxidant defense systems important for the reduction of oxidative stress induced by DNBS. 

## 5. Conclusions

**Figure 9:**
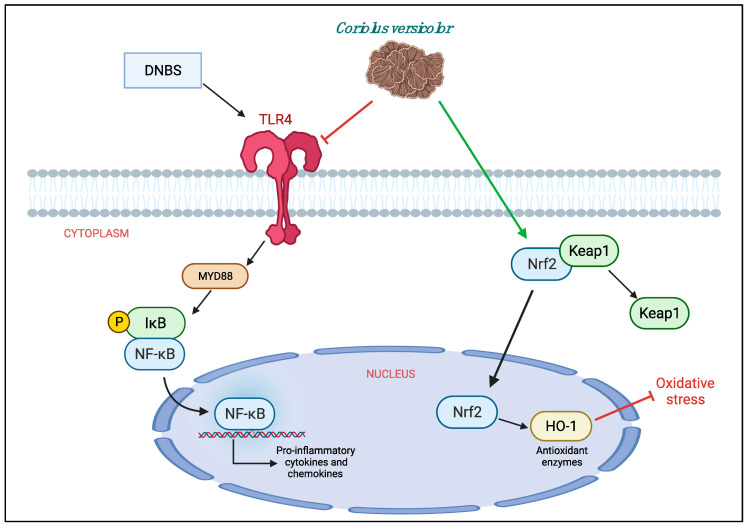
Protective effects of *Coriolus versicolor* on different pathways.

In conclusion, we can say that *CV* has shown to have both anti-inflammatory and antioxidant roles in the model of colitis induced by DNBS. Therefore, it could be used as a dietary supplement for the treatment of IBD. However, further studies are needed to define the mechanism of action well. Being a natural compound with both anti-inflammatory and antioxidant properties, it would be interesting to understand if its beneficial action is really related to TLR4 or if it acts through other pathways such as Nrf2 (Figure 9).

## Data Availability

For a rule of our laboratory the datasets used in the current study are available from the corresponding author (dipaolar@unime.it) on reasonable request.
