# Correction: Impellizzeri et al. Coriolus Versicolor Downregulates TLR4/NF-κB Signaling Cascade in Dinitrobenzenesulfonic Acid-Treated Mice: A Possible Mechanism for the Anti-Colitis Effect. *Antioxidants* 2022, *11*, 406

**DOI:** 10.3390/antiox14080925

**Published:** 2025-07-29

**Authors:** Daniela Impellizzeri, Roberta Fusco, Tiziana Genovese, Marika Cordaro, Ramona D’Amico, Angela Trovato Salinaro, Maria Laura Ontario, Sergio Modafferi, Salvatore Cuzzocrea, Rosanna Di Paola, Vittorio Calabrese, Rosalba Siracusa

**Affiliations:** 1 Department of Chemical, Biological, Pharmaceutical and Environmental Sciences, University of Messina, 98166 Messina, Italy; dimpellizzeri@unime.it (D.I.); tiziana.genovese@unime.it (T.G.); rdamico@unime.it (R.D.); rsiracusa@unime.it (R.S.); 2 Department of Clinical and Experimental Medicine, University of Messina, 98125 Messina, Italy; rfusco@unime.it; 3 Department of Biomedical, Dental and Morphological and Functional Imaging, University of Messina, 98125 Messina, Italy; marika.cordaro@unime.it; 4 Department of Biomedical and Biotechnological Sciences, University of Catania, 95125 Catania, Italy; trovato@unict.it (A.T.S.); marialaura.ontario@ontariosrl.it (M.L.O.); sergio.modafferi@studium.unict.it (S.M.); calabres@unict.it (V.C.); 5 Department of Pharmacological and Physiological Science, Saint Louis University School of Medicine, Saint Louis, MO 63104, USA; 6 Department of Veterinary Science, University of Messina, 98168 Messina, Italy

In the original publication [1], there was an error in the published **Figure 3G**. Nrf2 and NFkB were detected on the same membrane so they have the same laminin as a control. In fact, among the original bands sent to the journal during submission we show only one laminine. The error occurred in the construction of Figure 3G as the laminin band was overturned by mistake. The correct Figure 3G appears below. The authors state that the scientific conclusions are not affected. This correction has been approved by the Academic Editor. The original publication has also been updated.

## Figures and Tables

**Figure 3:**
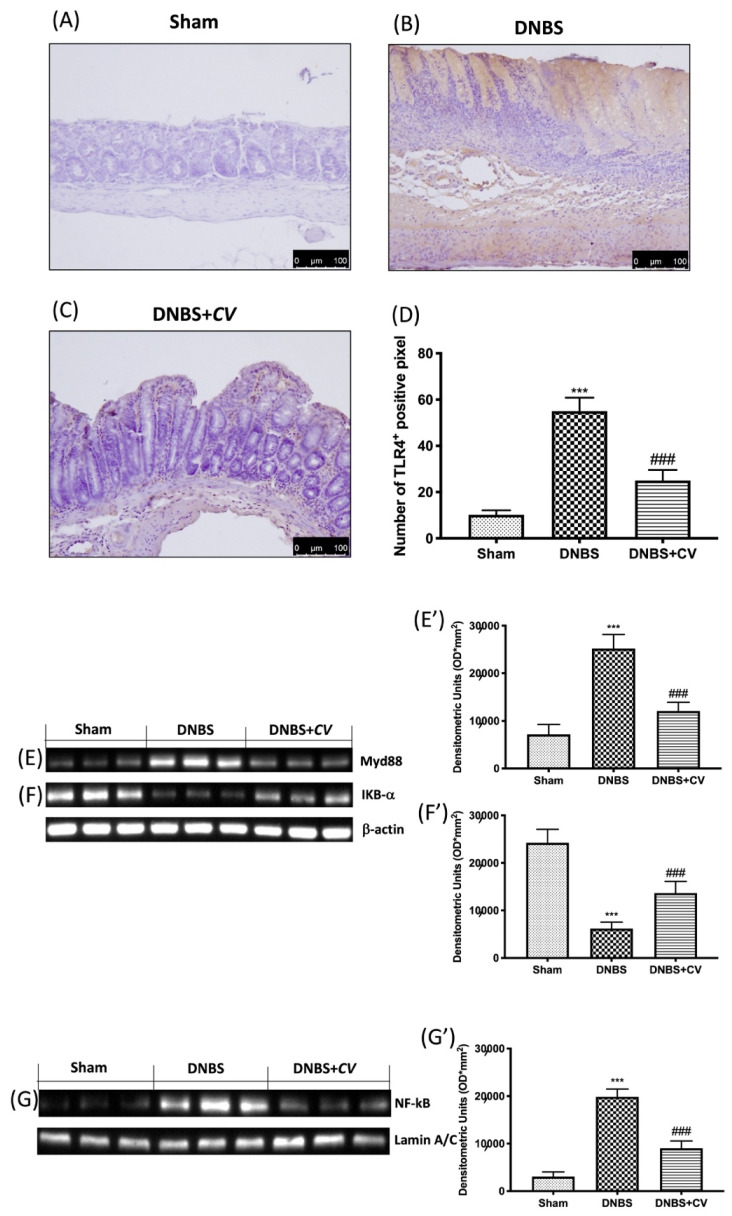
The effects of *CV* on TLR4, Myd88 and NF-kB pathway expression after DNBS-injection. Immunohistochemistry for TLR4 was evaluated in Sham (**A**), DNBS (**B**) and DNBS+*CV* (**C**). The results are expressed as number of TLR4^+^ positive pixel (**D**). Images are figurative of at least three independent experiments. Western blots for Myd88, IKB-α, and NF-kB. Representative Western blots for cytoplasmic Myd88 (**E**,**E’**), IKB-α degradation (**F**,**F’**), nuclear NF-kB translocation (**G**,**G’**) expression were performed. A demonstrative blot of lysates (5 animals/group), with a densitometric analysis for all animals, is shown (**E**’,**F’**,**G’**). Laminin was used as a control for normalization of NF-κB and Nrf2, which were incubated on the same membrane that was stripped by agitation with 3% glycine, pH 2, blocked in 5% non-fat dried milk/PBS for 1 hour at room temperature, and reincubated with the antibody. Values = means ± SD of 5 animals in each group. *** *p* < 0.001 vs. Sham; ### *p* < 0.001 vs. DNBS.
